# Simultaneous Detection of Both Single Nucleotide Variations and Copy Number Alterations by Next-Generation Sequencing in Gorlin Syndrome

**DOI:** 10.1371/journal.pone.0140480

**Published:** 2015-11-06

**Authors:** Kei-ichi Morita, Takuya Naruto, Kousuke Tanimoto, Chisato Yasukawa, Yu Oikawa, Kiyoshi Masuda, Issei Imoto, Johji Inazawa, Ken Omura, Hiroyuki Harada

**Affiliations:** 1 Oral and Maxillofacial Surgery, Graduate School of Medical and Dental Sciences, Tokyo Medical and Dental University, Tokyo, Japan; 2 Bioresource Research Center, Tokyo Medical and Dental University, Tokyo, Japan; 3 Hard Tissue Genome Research Center, Tokyo Medical and Dental University, Tokyo, Japan; 4 Department of Stress Science, Institute of Biomedical Sciences, Tokushima University Graduate School, Tokushima, Japan; 5 Genome Laboratory, Medical Research Institute, Tokyo Medical and Dental University, Tokyo, Japan; 6 Department of Molecular Cytogenetics, Medical Research Institute and School of Biomedical Science, Graduate School of Medicine, Tokyo Medical and Dental University, Tokyo, Japan; 7 Department of Human Genetics, Institute of Biomedical Sciences, Tokushima University Graduate School, Tokushima, Japan; 8 Oral Cancer Center, Tokyo General Hospital, Tokyo, Japan; Indiana University School of Medicine, UNITED STATES

## Abstract

Gorlin syndrome (GS) is an autosomal dominant disorder that predisposes affected individuals to developmental defects and tumorigenesis, and caused mainly by heterozygous germline *PTCH1* mutations. Despite exhaustive analysis, *PTCH1* mutations are often unidentifiable in some patients; the failure to detect mutations is presumably because of mutations occurred in other causative genes or outside of analyzed regions of *PTCH1*, or copy number alterations (CNAs). In this study, we subjected a cohort of GS-affected individuals from six unrelated families to next-generation sequencing (NGS) analysis for the combined screening of causative alterations in Hedgehog signaling pathway-related genes. Specific single nucleotide variations (SNVs) of *PTCH1* causing inferred amino acid changes were identified in four families (seven affected individuals), whereas CNAs within or around *PTCH1* were found in two families in whom possible causative SNVs were not detected. Through a targeted resequencing of all coding exons, as well as simultaneous evaluation of copy number status using the alignment map files obtained via NGS, we found that GS phenotypes could be explained by *PTCH1* mutations or deletions in all affected patients. Because it is advisable to evaluate CNAs of candidate causative genes in point mutation-negative cases, NGS methodology appears to be useful for improving molecular diagnosis through the simultaneous detection of both SNVs and CNAs in the targeted genes/regions.

## Introduction

Nevoid basal cell carcinoma syndrome (NBCCS; OMIM 109400), also known as Gorlin syndrome or basal cell nevus syndrome, is an autosomal dominant disorder that predisposes affected individuals to developmental defects, including bifid ribs and palmar or plantar pits, as well as tumorigenesis, including the development of basal cell carcinoma, medulloblastoma, or keratocystic odontogenic tumors (formerly known as odontogenic keratocysts) [1–3]. Heterozygous germline *PTCH1* mutations have been found in patients with Gorlin syndrome, and the spectrum of associated features is thought to arise from *PTCH1* malfunction [4, 5].

Diverse *PTCH1* mutations have been identified in Japanese Gorlin syndrome patients [6]. Although numerous germline *PTCH1* mutations have been reported, no clusters of mutations have been identified [6, 7]. Despite exhaustive analysis, *PTCH1* mutations are often unidentifiable in some patients [6]; for example, Sanger sequencing of *PTCH1* exons 2–23, encompassing the entire coding region, detected mutations in approximately 40%–70% of individuals with a typical clinical presentation of Gorlin syndrome [8, 9]. This failure to detect mutations is presumably because of copy number alterations (CNAs), including gross deletions, within or around the exons of *PTCH1*, or mutations of *PTCH1* outside the analyzed regions such as those within introns or regulatory elements. In addition, mutations in rare causative genes other than *PTCH1*, such as *PTCH2* [10, 11] and *SUFU* [12], have also been detected in individuals suffering from Gorlin syndrome.

Because mutations, or single nucleotide variations (SNVs), as well as CNAs of *PTCH1* and other genes related to the Hedgehog signaling pathway-related genes with numerous exons can be responsible for Gorlin syndrome, we conducted hybridization and polymerase chain reaction (PCR)-based next generation sequencing (NGS) analyses in a cohort of Gorlin syndrome-affected individuals from six unrelated families [13, 14] for a combined screening of causative alterations in candidate genes.

## Materials and Methods

### Patients and DNA samples

**Fig 1:**
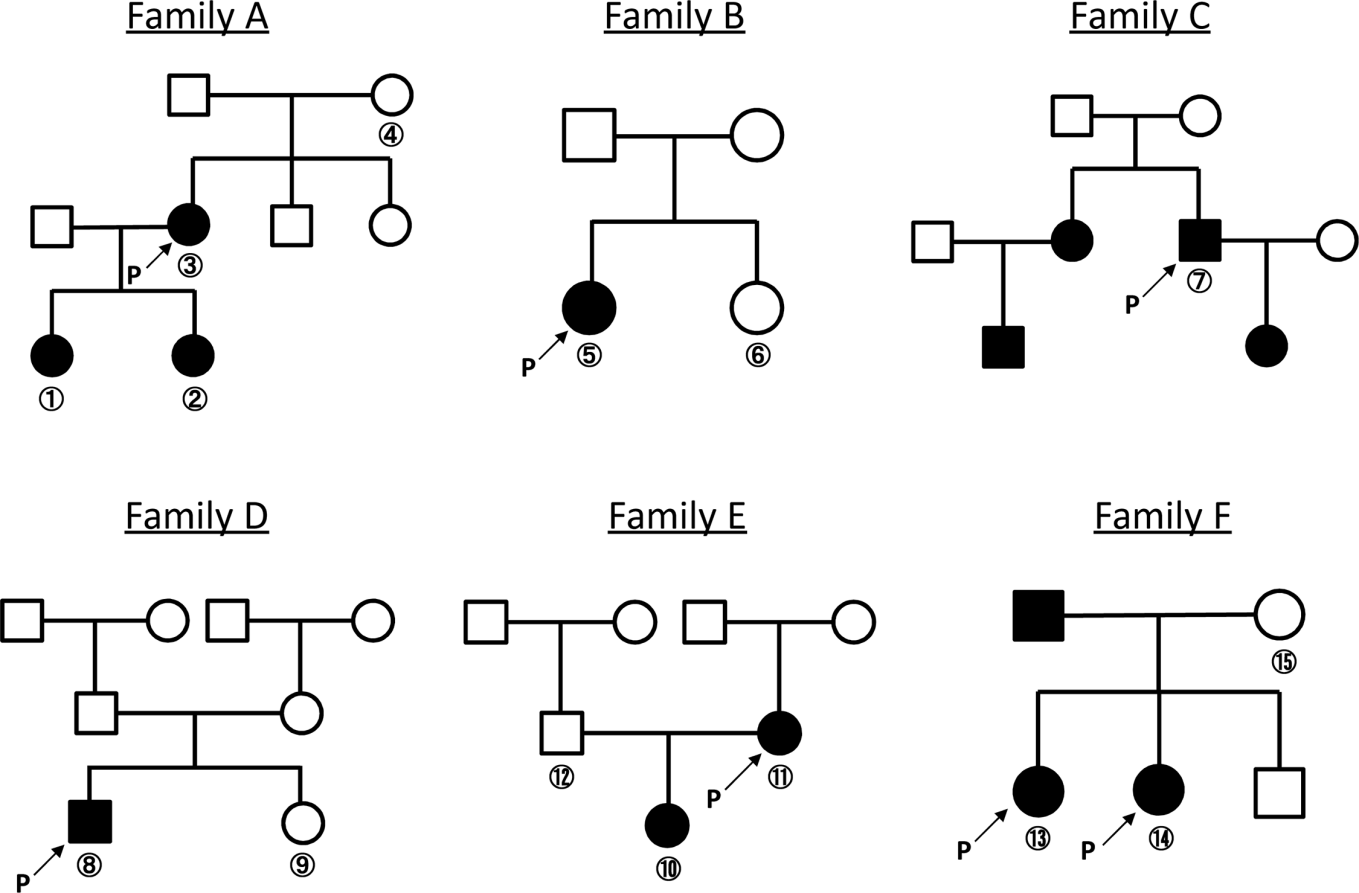
Family trees of the analyzed families affected by Gorlin syndrome. The shaded circles and squares represent affected individuals. Arrows with “P” represent the probands in each family.

We investigated 15 clinically evaluated Japanese individuals from six pedigrees, including 10 patients with Gorlin syndrome and five unaffected family members (Fig 1). Our group previously reported the clinicopathologic manifestations of all patients [13, 14], and this information is included in Table 1 with the corresponding patient identification codes.

Peripheral blood samples for study were collected after obtaining informed consent from all participants or their legal guardians, and genomic DNA was extracted using Wizard^®^ Genomic DNA Purification Kits (Promega, Madison, WI). The study was approved by the ethical committees of Tokyo Medical and Dental University and Tokushima University. For those agreeing to participate in this study, written consent was obtained from patients 20 years of age or older, and from the parent of minor children.

**Table 1:** Patients and unaffected family members.

Family ID	Individual No.	Patient / Unaffected member	Age	Gender	Symptoms agree with Kimonis criteria	Other symptoms	JOPM* No.	JOPM* Family ID	PLOS** No.	PLOS** Family ID
A	1	Patient	13	F	KCOT, Rib anomaly, Skin pits, Thoracic deformity	-	9	8	26	3
	2	Patient	10	F	KCOT, Rib anomaly, Skin pits, Ovarian fibroma	Ovarian cyst	10	8	25	3
	3	Patient	35	F	BCC, KCOT, Rib anomaly, Skin pits, Calcification of the falx cerebri	Multiple nevi	11	8	27	3
	4	Unaffected	-	F				8		3
B	5	Patient	10	F	KCOT, Rib anomaly, Skin pits, Vertebrate anomaly,	Dermoid cyst (skin), Glioma, Mandibular protrusion, Hydrocephalus, Psychomotor retardation, Macrocephaly	2	2		
	6	Unaffected	-	F				2		
C	7	Patient	65	M	BCC, KCOT, Skin pits	Multiple nevi, Epilepsy	15	10	36	-
D	8	Patient	15	M	KCOT, Rib anomaly, Skin pits, Calcification of the falx cerebri	Multiple nevi, Mandibular protrusion	6	6		
	9	Unaffected	-	F				6		
E	10	Patient	10	F	KCOT, Rib anomaly, Skin pits, Calcification of the falx cerebri	Language development disorder, Ventricular distention	18	12	23	2
	11	Patient	13	F	KCOT, Skin pits, Calcification of the falx cerebri	Unknown	19	12	24	2
	12	Unaffected	-	M				12		2
F	13	Patient	13	F	KCOT, Rib anomaly, Ocular hypertelorism	Multiple nevi, Mental retardation, Agenesis of corpus callosum, Prolonged retention of deciduous tooth	12	9		
	14	Patient	12	F	KCOT, Thoracic deformity	Multiple nevi, Prolonged retention of deciduous tooth	13	9		
	15	Unaffected	-	F				9		

KCOT, keratocystic odontogenic tumors.

* Reference No.13

** Reference No. 14.

### Design of custom targeted exome for NGS

A custom HaloPlex panel (Agilent Technologies, Santa Clara, CA) was designed using Agilent’s SureDesign tool (www.agilent.com/genomics/suredesign) to capture all exons with 25 bp of the flanking intronic sequences for eight genes in the Hedgehog signaling pathway: *PTCH1*, *PTCH2*, *SHH*, *SUFU*, *SMO*, *GLI*, *GLI2*, and *GLI3*. The entire custom design comprised 119 targets with a total size of 42.893 Kb, 3 283 total amplicons, and a final probe design size of 80.612 Kb, with a total theoretical coverage of 99.98% for the targeted regions.

### Sequence capture and NGS

Sequence capture was performed using the HaloPlex Target Enrichment System for Ion Torrent sequencing according to the manufacturer’s instructions (Protocol Version D, Agilent Technologies). Pooled samples for multiplex sequencing were sequenced using the Ion Personal Genome Machine System (Thermo Fisher Scientific, Waltham, MA) with 318 chips (Thermo Fisher Scientific).

### SNV analysis and validation

**Fig 2:**
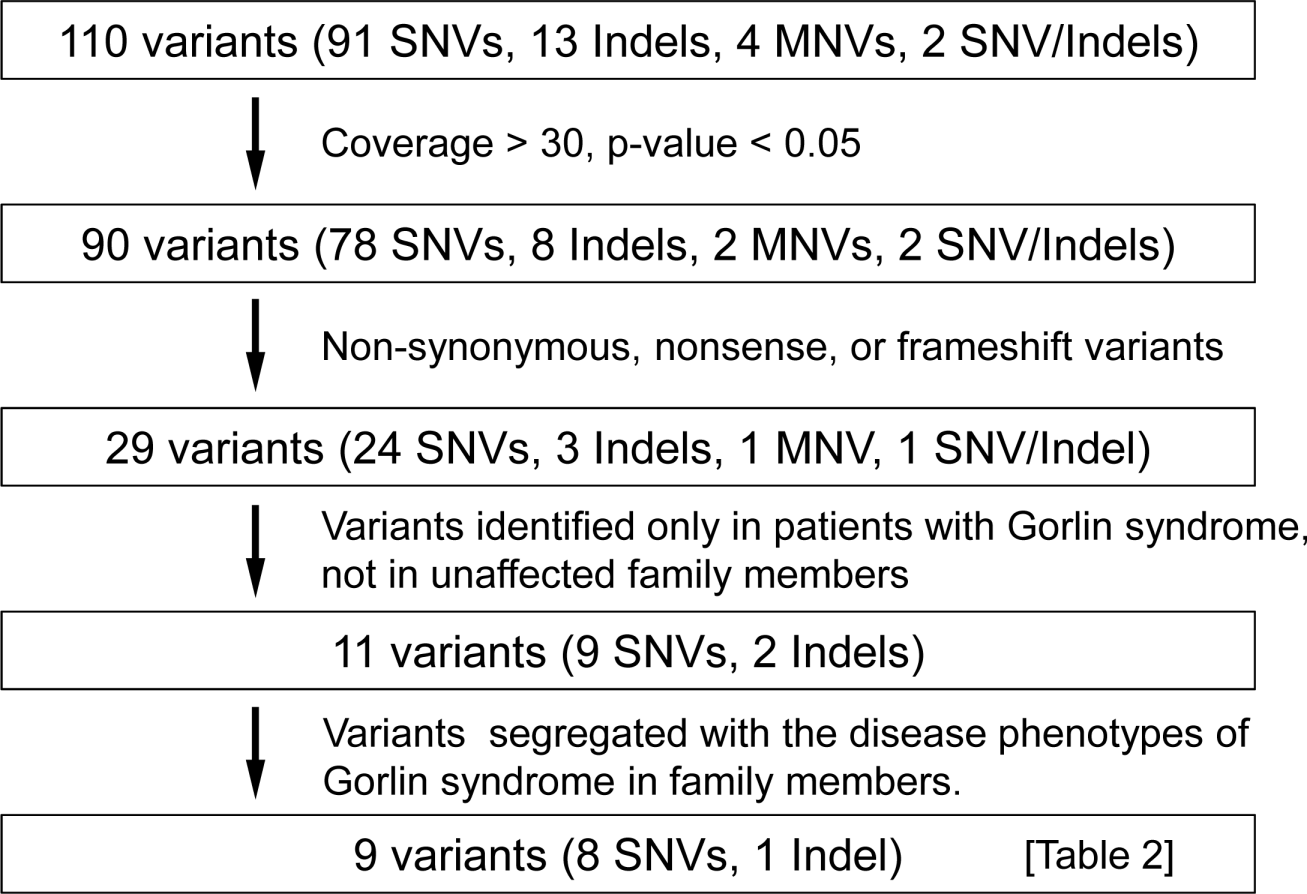
Flow chart indicating the validation process for variants. After four validation steps, nine genetic variants were selected, as shown in Table 2.

Data were analyzed using Torrent Suite Software v4.2.1 (Thermo Fisher Scientific) and Ion Reporter Software v4.6 (Thermo Fisher Scientific). The flow chart used to select candidate genes is shown in Fig 2. Annotated variants were selected according to the criterion that the causal variants should be segregated in patients with Gorlin syndrome-affected family members. Candidates for pathogenic variants within the *PTCH1* were confirmed by Sanger sequencing. The pathogenicity of missense variants was assessed using tools for prediction of possible impact of an amino acid substitution on the structure and function of a human protein, such as Sorting Intolerant From Tolerant (SIFT) v5.1.1 (http://sift.bii.a-star.edu.sg/), PolyPhen-2 v2.2.2r398 (http://genetics.bwh.harvard.edu/pph2/index.shtml), MutationTaster (http://www.mutationtaster.org/index.html). SIFT examines the degree of conservation for amino acid residues across species, and calculated probabilities are used to partition ‘tolerated’ (normalized probability >0.05) from ‘damaging’ (normalized probability ≤0.05) substitutions for prediction of a phenotypic effect. PolyPhen-2 finds change in protein structure and function, and provides both a qualitative prediction (one of 'probably damaging', 'possibly damaging', 'benign' or 'unknown') and a score. MutationTaster checks evolutionary conservation, change in protein structure and function, and additionally effects on splicing or mRNA expression, and predicts the disease potential: ‘disease causing’, which is probably deleterious, ‘disease causing automatic’, which is known to be deleterious, ‘polymorphism’, which is probably harmless, and ‘polymorphism automatic’, which is known to be harmless. Identified SNVs was also evaluated by comparing with known alterations reported in mutation databases, such as the Human Gene Mutation Database (HGMD professional 2015.2, http://www.hgmd.cf.ac.uk/ac/index.php), and ClinVar (http://www.ncbi.nlm.nih.gov/clinvar/).

### CNA analysis and validation

The coverage of every target region in each sample was normalized and compared to the average normalized data from all samples from unaffected family members analyzed in the same run to determine the relative coverage ratio [15]. Deletions and duplications were suspected to be present within a genomic interval if this ratio fell below 0.7 or rose above 1.3, respectively. CNA validation and detailed mapping of altered regions were performed using an Affymetrix CytoScan HD chromosome microarray platform (Affymetrix, Santa Clara, CA), which provides 906 600 polymorphic (SNP) and 946 000 non-polymorphic (CNA) markers, according to the manufacturer’s recommendations. The raw data were processed using Chromosome Analysis Suite software (ChAS, Affymetrix), and the output data were interpreted with the UCSC Genome Browser (http://genome.ucsc.edu; GRCh37/hg19 assembly) [16].

## Results

In the NGS analysis, which applied our customized Hedgehog pathway-related gene panel to our cohort of Gorlin syndrome patients and their unaffected family members, a mean coverage of 282X was obtained per sample and target region. We observed an average of 92.48% of bases at >1X coverage, 81.68% of bases at >20X coverage, and 64.62% of bases at >100X coverage. Specific SNVs causing inferred amino acid changes in *PTCH1*, *PTCH2*, *and GLI2* were found in four, one, and one families (seven, one, and one affected individuals), respectively, whereas CNAs within or around *PTCH1* were found in two families in which no possible causative SNVs were detected (Table 2).

**Table 2:** SNVs identified in affected patients but not in unaffected family members.

Family ID	Indivisual No.	Gene		Mutation									
	(Affected vs. unaffected)			Nucleic acid change	Type	AA cange	Exon	SIFT prediction (score)	PolyPhen-2 prediction (score)	MutationTaster prediction	Frequency in HGVD	dbSNP	Reported as pathogenic alteration
A	1–3 vs. 4	*PTCH1*	NM_000264.3(PTCH1_v001)	c.1502A>G	Missense	p.Q501R	10	Damaging (0.00)	Probably damaging (0.967)	Disease_causing	-	-	-
		*PTCH1*	NM_000264.3(PTCH1_v001)	c.2222C>T	Missense	p.A741V	14	Damaging (0.01)	Possibly damaging (0.787)	Disease_causing	0.004	rs2227971	-
		*PTCH1*	NM_000264.3(PTCH1_v001)	c.3953C>T	Missense	p.P1318L	23	Tolerated (0.13)	Benign (0.014)	Disease_causing	0.0001	-	-
B	5 vs. 6	*PTCH1*	NM_000264.3(PTCH1_v001)	c.2619C>A	Nonsense	p.Y873*	16	-	-	-	-	-	Boutet et al., 2003
C	7	*PTCH1*	NM_000264.3(PTCH1_v001)	c.3394T>C	Missense	p.S1132P	20	Damaging (0.01)	Probably damaging (1.000)	Disease_causing	-	-	Reifenberger et al., 2001
		*PTCH2*	NM_001166292.1(PTCH2_v001)	c.221G>A	Missense	p.R74H	2	Damaging (0.01)	Possibly damaging (0.933)	Disease_causing	0.006	-	-
		*PTCH2*	NM_001166292.1(PTCH2_v001)	c.524G>T	Missense	p.R175L	4	Tolerated (0.11)	Probably damaging (0.990)	Disease_causing	0.006	-	-
D	8 vs. 9	*PTCH1*	NM_000264.3(PTCH1_v001)	c.1591_1601del	Frameshift	p.I531Gfs*92	9	-	-	-	-	-	-
		*GLI2*	NM_005270.4(GLI2_v001)	c.1906G>C	Missense	p.A636P	11	Tolerated (0.26)	Possibly damaging (0.877)	Polymorphism	-	-	-
E	10,11 vs. 12	No											
F	13,14 vs. 15	No											

### Causative genetic alterations identified in each family

#### Family A

In family A, we identified three mutations in *PTCH1* that had been inferred to cause amino acid changes (Table 2). Among these, the p.Q501R and p.A741V changes in *PTCH1* were predicted to be functionally pathogenic *in silico*, but have not been reported in either HGMD or ClinVar. Because p.A741V is listed in the Human Genetic Variation Database (HGVD, http://www.genome.med.kyoto-u.ac.jp/SnpDB/index.html) at a low frequency (allele frequency = 0.004) and in dbSNP (http://www.ncbi.nlm.nih.gov/SNP/, rs2227971), we considered it a non-causative mutation with respect to Gorlin syndrome. In addition, p.Q501R mutation occurred right next to the 4th transmembrane domain (p.502–522). In HGMD, 26 and 2 of 53 missense mutations reported as disease-causing mutations locate within and right next to any of twelve transmembrane domains of PTCH1, respectively. Since only 251 and 24 of 1447 amino acids locate within or right next to transmembrane domains, transmembrane domains of the PTCH1 protein seem to be hotspots for missense mutations contributing to functional defects. Therefore, we concluded that p.Q501R was a novel missense mutation causative for Gorlin syndrome in family A.

#### Family B

In family B, we identified a nonsense mutation in *PTCH1* (p.Y873*). As this mutation was previously reported to be causative for NBCCS by Boutet et al. [17], we concluded that this alteration was responsible for Gorlin syndrome in family B.

#### Family C

In family C, we identified missense mutations in *PTCH1* and *PTCH2* (Table 2). The p.R175L mutation in *PTCH2* was predicted to be “Tolerated” by SIFT, and both p.R175L and p.R74H mutations in *PTCH2* were observed in the HGVD at low frequencies (allele frequency = 0.006 for both variations), suggesting those alterations to be rare benign variants. As the p.S1132P mutation of *PTCH1* was reported to be causative for NBCCS [18], we concluded that this alteration was responsible for Gorlin syndrome in family C.

#### Family D

In family D, we identified a possibly deleterious frameshift mutation in *PTCH1* (p.I531Gfs*92) resulting in a truncated protein lacking 8 of 12 transmembrane domains that had not been reported in HGMD or ClinVar. On the other hand, the p.A636P mutation in *GLI2* was predicted as “Tolerated” and a “Polymorphism” by SIFT and MutationTaster, respectively. Accordingly, we concluded that p.I531Gfs*92 was a novel *PTCH1* mutation causative of Gorlin syndrome in family D.

#### Families E and F

**Fig 3:**
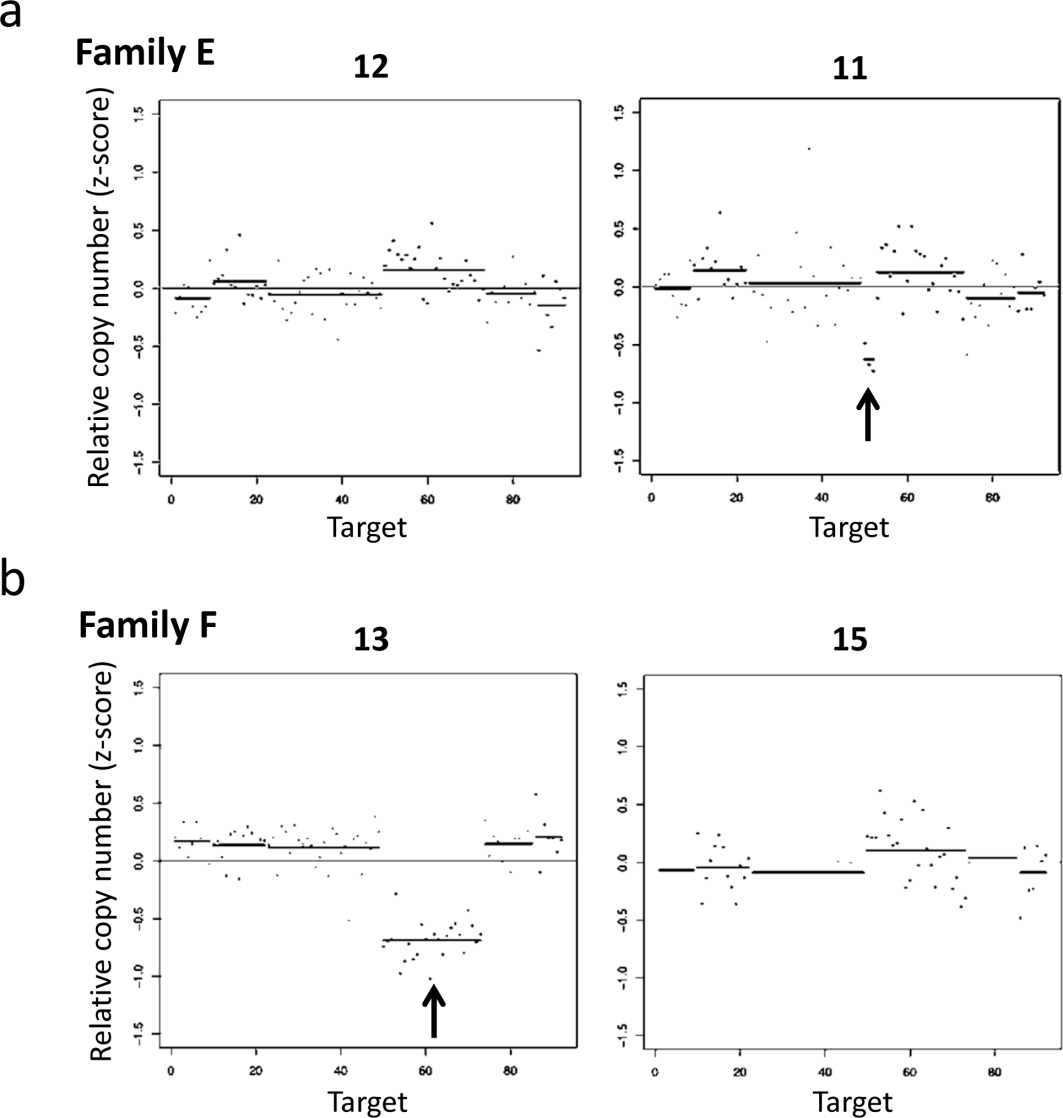
Graphical representations of DNA copy number alterations across target regions (1p33-p34, 2q14, 7p13, 7q32.3, 7q36, 9q22.3, 10q24.32, and 12q13.2-q13.3), studied via targeted NGS-based exome analysis. (a) The copy number loss shows a region from exon 22 to the 3'-UTR of *PTCH1* in a Gorlin syndrome patient (right panel; individual No. 11, Family E), but not in an unaffected family member (left panel; individual No. 12, Family E). (b) The copy number loss shows a sequencing region of 9q22.3 in a Gorlin syndrome patient (left panel; individual No. 13, Family F), but not in an unaffected family member (right panel; individual No. 15, Family F).

**Fig 4:**
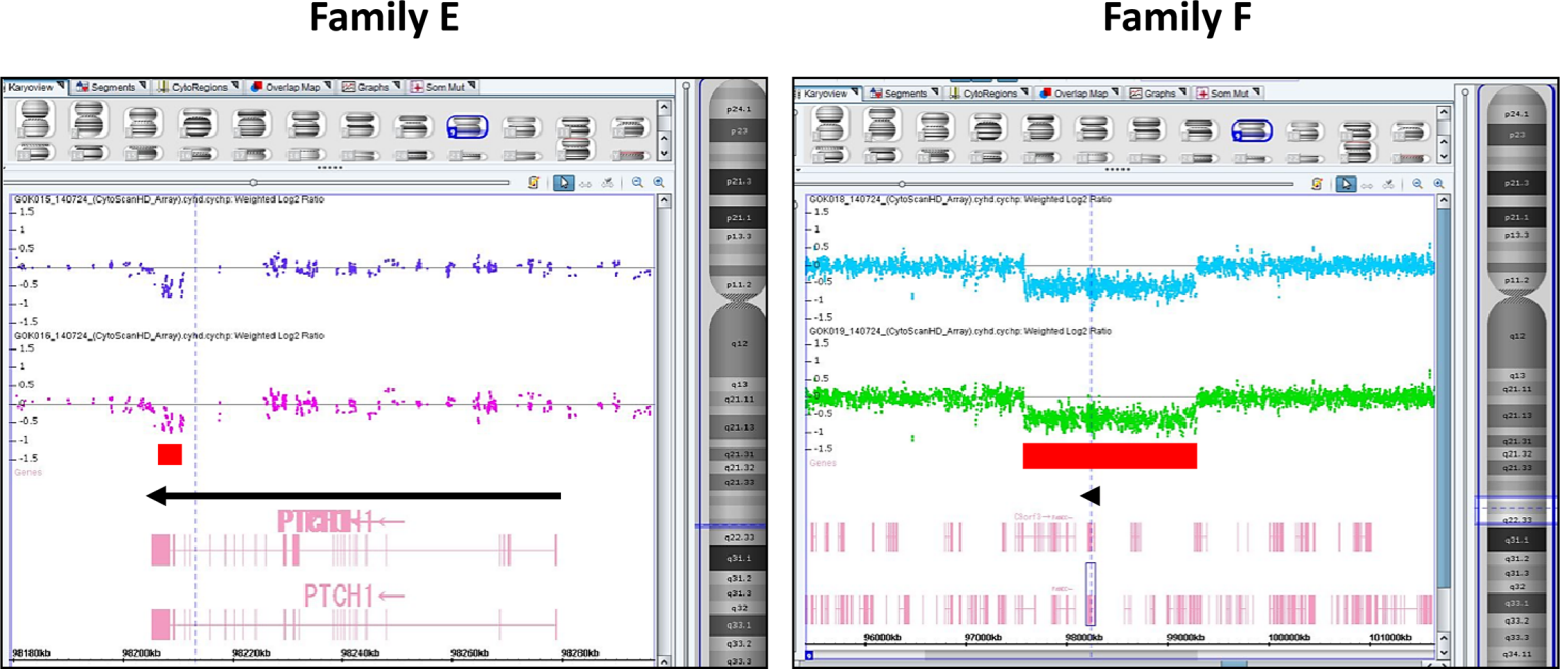
Image of deleted region detected by an array-based method using an Affymetrix CytoScan HD chromosome microarray platform. Left panel: The deletion size was approximately 3.8 kb within the *PTCH1* (from exon 23 to part of the 3’-UTR) in family E (upper and lower lanes are individual No. 10 and No. 12, respectively). Right panel: A relatively large deletion (approximately 1.7-Mb) containing genes from *C9orf3* to *CDC14B*, including full-length *PTCH1*, was detected in family F (upper and lower lanes are individual No. 13 and No. 14, respectively).

In families E and F, we were unable to identify any causative SNVs within the target gene coding regions, but rather found deletions within the *PTCH1* gene (Fig 3). A deletion from exon 22 to the 3'-UTR of *PTCH1* was observed in family E (Fig 3A), whereas all exons of *PTCH1* were deleted in family F (Fig 3B). These deletions were confirmed using an array-based copy number analysis (Fig 4). The size of the deletion in family E, as determined by an array-based method, was approximately 3.8 kb (chr9:98,207,091–98,210,940) within *PTCH1* (from exon 23 to the part of 3’-UTR). As informative probes were not arranged around exons 21 and 22 on the Affymetrix CytoScan HD array platform, it is possible that the deleted region was underestimated by array-based methods relative to the NGS-based method. In family F, a relatively large deletion (approximately 1.7 Mb) (chr9:97,579,146–99,280,739) containing several genes from *C9orf3* to *CDC14B*, including full-length *PTCH1*, was detected. These alterations have not been previously reported in HGMD or ClinVar, indicating them to be novel deletions causative of Gorlin syndrome. The array-based karyotype results of patients with Gorlin syndrome in families E and F were arr[hg19] 9q22.32(98,207,091–98,210,940)×1 and arr[hg19] 9q22.32(97,579,146–99,280,739)×1, respectively.

Collectively, the Gorlin syndrome phenotypes were consistent with heterozygous PTCH1 defects caused by various types of mutations or deletions in all investigated families with this disease.

## Discussion

Numerous germline *PTCH1* mutations, including nonsense, missense, frameshift, and splicing mutations as well as gross insertions and deletions, have been reported in Gorlin syndrome patients. Miyashita et al. (http://www.med.kitasato-u.ac.jp/∼molgen/sub10.html) genotyped a panel of Gorlin syndrome patients, finding *PTCH1* mutations within various percentages of the patient group: 52.4% were frameshift, 14.3% were nonsense, 11.1% were splicing, and 9.5% were missense mutations. Despite exhaustive analysis, *PTCH1* mutations could not be identified in some patients. This failure to detect mutations was presumably due to large deletions or the existence of mutations outside of the regions analyzed or in genes other than *PTCH1* [6]. Although mutations in *PTCH2* and *SUFU* had been previously implicated in Gorlin syndrome, none of the prior studies that comprehensively analyzed the Hedgehog signaling pathway-related genes identified causative genes other than those three genes.

In this study, we used NGS to simultaneously screen all exons of 8 Hedgehog signaling pathway genes in 15 individuals from 6 families, including Gorlin syndrome patients and their unaffected family members. Through targeted resequencing of all coding exons with flanking exon-intron junctions, as well as simultaneous evaluation of the copy number status using alignment map files obtained via NGS, we found that Gorlin syndrome phenotypes could be explained by *PTCH1* mutations or deletions in all affected patients. The p.Y873* and p.S1132P mutations of *PTCH1* were previously reported to be causative for NBCCS [17, 18]. Another missense mutation, a small deletion, and two gross deletions were newly identified alterations possibly responsible for Gorlin syndrome in the affected families. Although the p.P1318L *PTCH1* mutation was previously reported by our group [14], we concluded this mutation not to be causative because it was predicted to be functionally “tolerated” by SIFT and have been reported in HGVD as a rare variation. Those results suggest that an NGS-based approach to the simultaneous detection of candidate for causative alterations in putative target genes with *in silico* annotation could effectively identify genetic alterations responsible for Gorlin syndrome. Possibly disease-causing alteration was detected in all cases tested in this study. However, if mutations existed outside of regions targeted for NGS analysis, they would be missed through this approach. Further examinations using larger cohorts containing both familial and sporadic cases will be needed to verify the validity of this approach.

Despite reports of numerous germline *PTCH1* mutations in patients with Gorlin syndrome, no clustering of mutations has been identified [7]. Previous studies revealed a lack of genotype–phenotype correlations between mutations in *PTCH1* and the major clinical features of Gorlin syndrome [8, 19]. On the other hand, large deletions containing the *PTCH1* might cause distinguishing symptoms such as mental retardation [20, 21] because of the deletion of other genes included in the regions around *PTCH1*. In our study, the 1.7-Mb gene deletion detected in patient 13 from family F, who exhibited mental retardation, included *PTCH1* and seven other Refseq genes: *C9orf3* (NM_032823), *FANCC* (NM_001243743), *ERCC6L2* (NM_001010895), *HSD17B3* (NM_000197), *SLC35D2* (NM_007001), *HABP4* (NM_014282), and *CDC14B* (NM_003671). Notably, among these, hyaluronan binding protein 4 (HABP4), encoded by *HABP4*, is known to bind to FXR1 (fragile X mental retardation-related protein 1),[22] suggesting that *HABP4* haploinsufficiency due to heterozygous deletion was involved in the observed pathogenesis (i.e., mental retardation) observed in the Gorlin syndrome patient from family F. This haploinsufficiency might have disrupted the interaction of HABP4 with FXR1, although only one of the two affected family members clearly exhibited this phenotype.

In summary, we systematically screened individuals with familial Gorlin syndrome for causative genetic alterations using hybridization and PCR-based NGS, and found that various *PTCH1* mutations, including large deletions, could explain the disease phenotypes. Because it is advisable to examine the CNAs of candidate causative genes in point mutation-negative cases, NGS methodology appears to be useful for improving the molecular diagnosis of Gorlin syndrome through the simultaneous detection of both SNVs and CNAs in targeted genes/regions.
